# Effects of root restriction on phytohormone levels in different growth stages and grapevine organs

**DOI:** 10.1038/s41598-021-04617-6

**Published:** 2022-01-25

**Authors:** Jiajia Li, Dongmei Li, Boyang Liu, Ruiqi Wang, Yixuan Yan, Guanhan Li, Lei Wang, Chao Ma, Wenping Xu, Liping Zhao, Xiangyi Li, Shiping Wang

**Affiliations:** Department of Plant Science, School of Agriculture and Biology, Shanghai Jiao Tong University, Shanghai, China

**Keywords:** Plant hormones, Plant physiology

## Abstract

Phytohormones play important roles in germination, blossom, senescence, abscission of plants by a series of signal transduction and molecular regulation. The purpose of this research was to investigate the influence of root restriction (RR) cultivation on plant endogenous hormone variation tendency at different growth stages in diverse organs or tissues. ‘Muscat Hamburg’ (Vitis ‘Muscat of Alexandria’ × Vitis ‘Trollinger’) grapevine was used as test material. High Performance Liquid Chromatography (HPLC) was used to quantify hormone levels, qRT-PCR was used to quantify the expression of genes related to hormone biosynthesis pathway, and determined parameters of growth and photosynthetic, aiming to investigate the influence of root restriction on the formation and metabolism of phytohormones, as well as the degree of correlation between phytohormones and plant growth and photosynthetic intensity under root restriction. By measuring the photosynthetic rate of leaves at the stages of core-hardening, veraison and maturity, it was found that root restriction could reduce most photosynthetic parameters. The results also revealed that RR treatment increased abscisic acid (ABA), salicylic acid (SA), zeatin riboside (ZR), N6-(delta 2-isopentenyl)-adenine nucleoside (iPR) concentrations, while reduced auxin (IAA), 3-indolepropionic acid (IPA), 3-indolebutyric acid (IBA), gibberellin A_3_ (GA_3_), zeatin (ZT), N6-(delta 2-Isopentenyl)-adenine (iP), kinetin (KT), jasmonic acid (JA) and methyl jasmonate (MeJA) concentrations in most organs and at most developmental stages. RT-qPCR was carried out to further explore the effect of root restriction on genes expression of ABA, SA and IAA biosynthesis pathways at molecular level. Meanwhile, through correlation analysis, we found that different phytohormones contributed differently to physiological indicators, there existed strong correlation of ABA, KT, MeJA, iPR, SA, JA with leaf photosynthesis, GA_3_, IBA, ZR, IAA, ZT with fruit quality. In addition, we also found that the shoot growth related parameters were closely correlated with JA, IPA and iP. To sum up, our results suggested that RR treatment could significantly increase soluble solid content, regulate the growth and photosynthesis of grapevine, by affecting the biosynthesis of phytohormones. It could further prove that root restriction was a feasible technique to ameliorate the phenomenon of low quality in grape berry in southern China.

## Introduction

Grape (*Vitis vinifera* L*.*), an important economic fruit variety widely cultivated around the world, could be produced for both fresh eating and wine making. With the development of berries, several changes of phenotypes take place including fruit expand, several changes of fruit quality parameters including soluble sugar content increase^1^, anthocyanin content increase as well as stresses and diseases resistance improve^2–5^. Such phenotypic alterations and metabolic changes are regulated by phytohormones including auxin (IAA)^6,7^, abscisic acid (ABA)^8–10^, ethylene (ETH)^11,12^, gibberellin (GA)^13^, salicylic acid (SA)^14,15^, cytokinin (CTK)^16^, and jasmonic acid (JA)^17,18^. Previous studies focusing on the signaling pathways and anabolism of endogenous hormones have revealed the transport mechanism successfully. For example, it had been found that *AUX/IAA* (auxin primary response gene) and *ARF* (plant morphogenesis key small G protein regulators) proteins could mediate both vascular bundle development and organogenesis^7^, it had also been demonstrated that the ubiquitin–proteasome pathway was closely related to IAA signaling^19^, and the degradation of this kind of protein was extremely vital for the fission of plant cells^20^. In several studies of ABA biosynthesis and signal transduction, it has been widely acknowledged that ABA synthesis is regulated by FDA (Fluorescein diacetate) modifications^21,25^, while carotenoids might also medicate its synthesis in higher plants^22^.In addition, previous studies focused on the CTKs biosynthesis and degradation have revealed that *STM* (Key genes for apex development) could regulate cytokinin accumulation^23^ and *KNOXI* (Key genes of meristem development and maintenance) could also activate cytokinin responses to cell fission^24^. Moreover, the anabolic pathways and transport mechanisms of other phytohormones (ETH, JA, SA, CTKs) in recent studies are more distinct^26–32^, indicating that the biosynthesis and metabolism of all phytohormones are regulated by a series of genes or enzymes, which are sophisticated and complex.

Current studies have proved that the level of endogenous hormones in plants was not only affected by the climate^33^, but also by the cultivation environment and irrigation regime^34^. For examples, researchers found the ABA and ethylene signal components were differently accumulated in two distinct climates by analysing the transcriptional expression patterns of carotenoid metabolism in ‘Cabernet Sauvignon’ (*Vitis vinifera* L.) grapes^35^, further study also revealed that regulated deficit irrigation (RDI) could significantly increase ABA levels in ‘*Cabernet Sauvignon’* (*Vitis vinifera* L.) wine grapes while reducing IAA levels^36^. However, researches on the effect of other cultivation patterns on endogenous hormone contents in grapevine are not clear now.

Root restriction (RR) is a widely used cultivation method which restrict the root system in a closed container in order to control the growth of roots, ultimately leading to the accumulation of sugar in berries and anthocyanin in pericarp^37^. In southern China, because of the hot and humid climate, the grape berries always attain a very low level of sweetness, but the root restriction cultivation technique breaks this limitation successfully. Previous studies have demonstrated that RR treatment could shorten shoot length^38^, increase total soluble solid (TSS) content^39^, regulate source-base balance as well as improve stress resistance ability^40^. It has been widely acknowledged that RR treatment could influence the formation and transportation of phytohormones, but the variation tendency during different stages in different organs are still poor to understand. A thorough research in exploring the variation tendencies of endogenous hormones including IAA, IBA, IPA, CTKs, GA, ABA, JA, MeJA was performed, and a thorough research in investigating correlation between phytohormones and grape physiological parameters was also necessary, above results will better understand the action mechanism and effect with RR treatment. Therefore, a total of 27 physiological parameters of ‘*Muscat Hamburg’ (Vitis ‘Muscat of Alexandria’* × *Vitis ‘Trollinger’)* grape were measured, 13 endogenous hormone concentrations during four growth stages (I: Budbreak, II: Blossom, III: Veraison, IV: Maturity) in different organs (bud, root, berry, flower, stem, leaf) were analyzed by HPLC, and ABA, IAA and SA biosynthesis related genes were quantified by qRT-PCR, aiming to investigate the impact of RR treatment on the concentrations of endogenous hormones. And through correlation analysis, ultimately inquired the relationship between root restriction and changes in physical parameters and plant endogenous hormone concentrations, as well as their relational degree.

## Results

### Analysis of different physiological parameters

**Figure 1:**
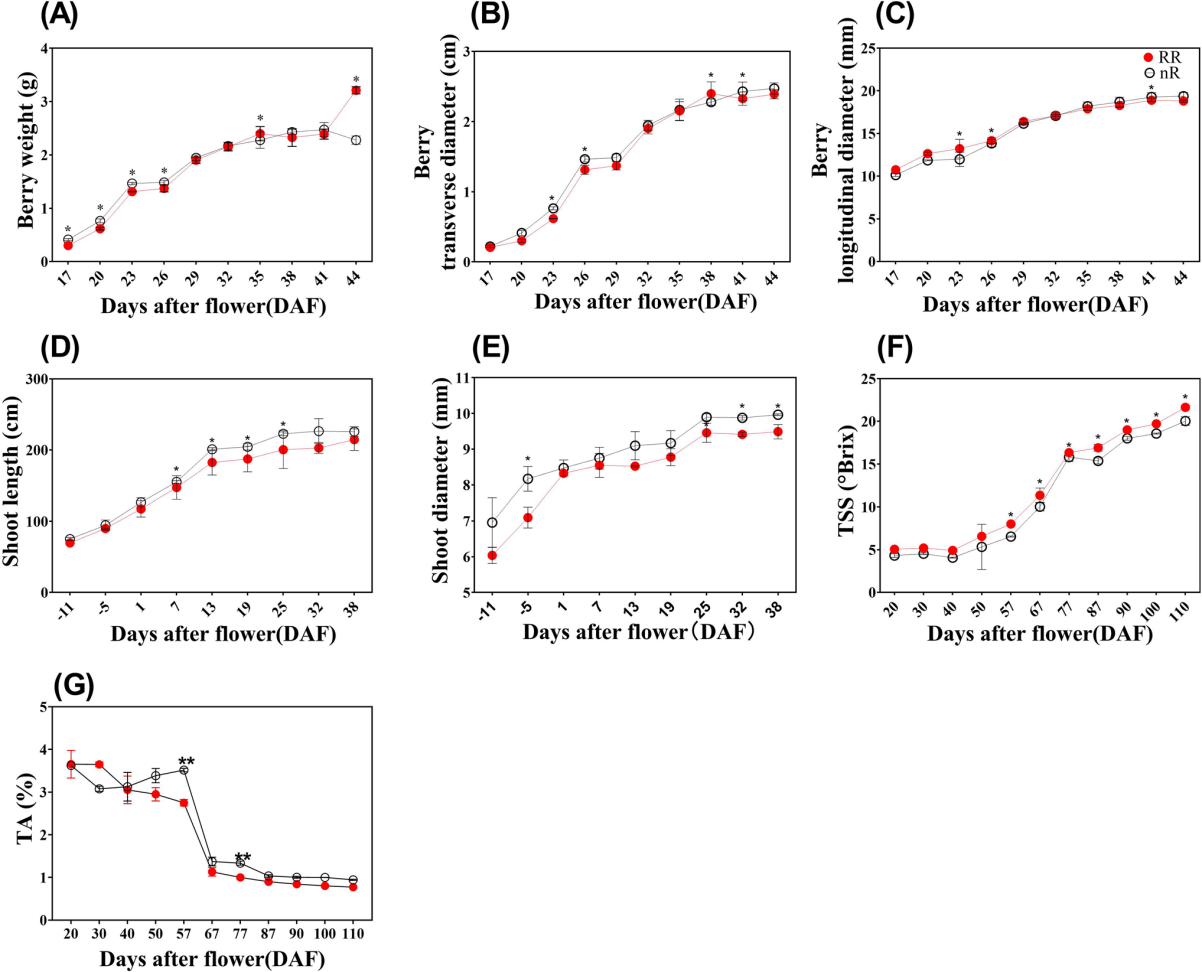
The variation tendencies of physiological parameters with RR treatment and control in veraison. **(A)** Berry weight, **(B)** Transverse diameter, **(C)** Longitudinal diameter, **(D)** Shoot length, **(E)** Shoot diameter, **(F)** Total soluble solid (TSS), **(G)** Titratable acid (TA) content. Data from the control group were presented as hollow black circles and data from the RR treatment were presented as solid red circles. nR indicated the control group, while RR indicated the root restriction group, * indicated the significant differences between treatments, while ** indicated extremely significant differences between treatments.

The variation tendencies of physiological parameters with RR treatment and control group during veraison were shown in the Fig. 1, Total eight physiological parameters with RR treatment and the control group were quantified. Berry weights with RR treatment were significantly higher than with the control group in DAF (Days after flower) 35 and 44 stages (Fig. 1A). The berry longitudinal diameters with RR treatment were significantly higher than (Fig. 1B,C) with the control group in DAF 23 stage as well as the parameter of transverse diameter in DAF 38 stage. Shoot lengths and diameters had the same variation trends, which displayed that with RR treatment would be lower than with the control group in DAF 7, 13, 19, 25 stages (Fig. 1D,E). To further investigate other physiological parameters, TSS, photosynthetic rate and stomatal conductance were also detected. Figure 1F well reported that there existed extremely significant differences in TSS between RR treatment and control group in DAF 57, 67, 77, 87, 90, 100, 110 stages. Meanwhile, titratable acid (TA) content, as an indicator of fruit taste, contrary to TSS, showed a decreasing trend with fruit ripening. In DAF 57 and DAF 77 stages, the TA contents in RR treatment group were significantly lower than that in nR treatment group, which also demonstrated the improvement of fruit taste (Fig. 1G).

### Analysis of photosynthetic indexes in three grapevine developmental stages

**Figure 2:**
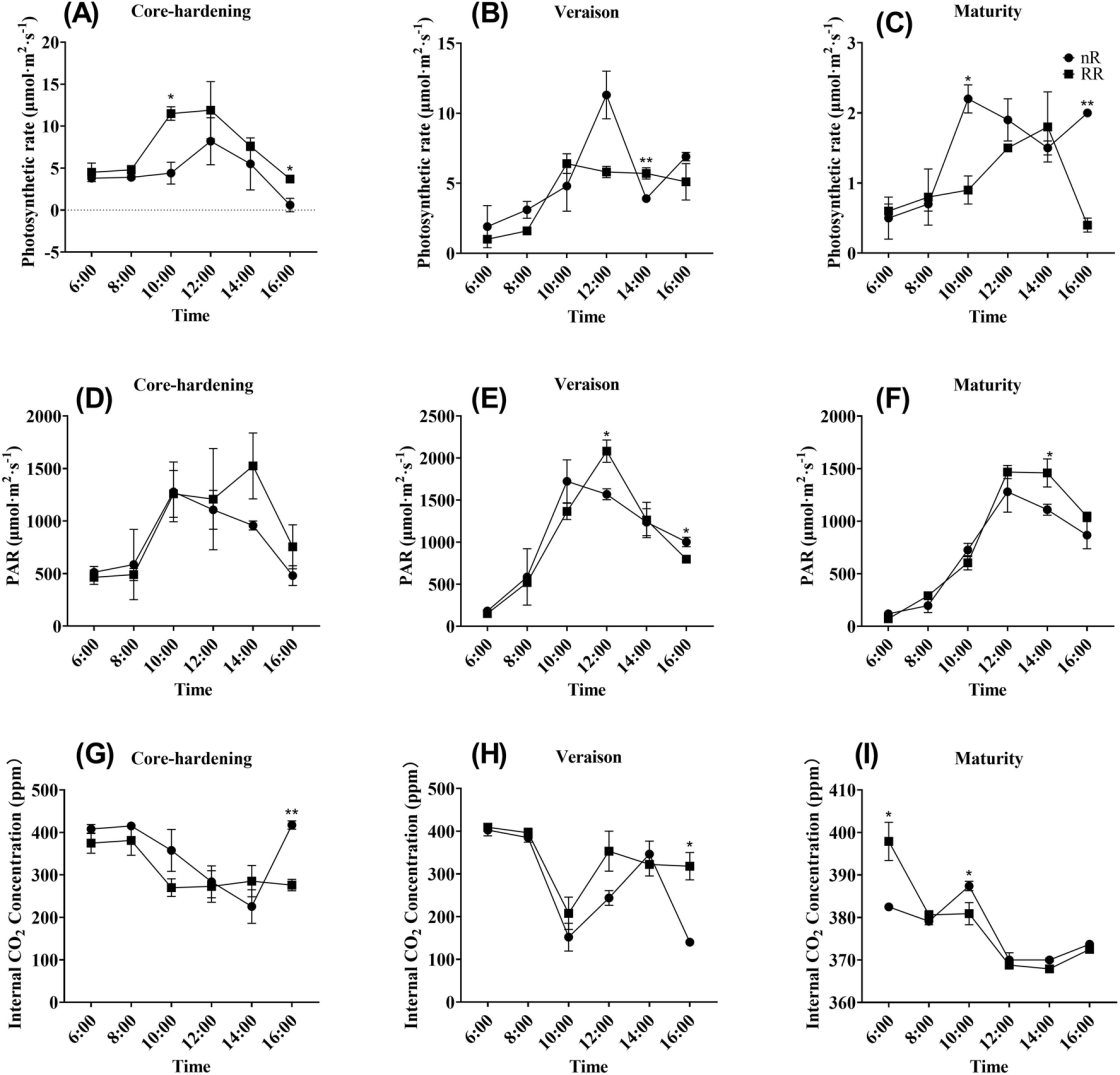
The variation tendencies of photosynthetic parameters with RR treatment and control in core-hardening, veraison and maturity stages. **(A–C)** Photosynthetic rate in three developmental stages; **(D–F)** PAR in three developmental stages; **(G–I)** Internal CO_2_ concentration in three developmental stages. Data from the control group were presented as circles and data from the RR treatment were presented as square. nR indicated the control group, while RR indicated the root restriction group, * indicated the significant differences between treatments, while ** indicated extremely significant differences between treatments.

Photosynthesis is an indispensable life activity for plant growth and development, for almost all organisms in the biological world, this process is the key to their survival. As results showed in the Fig. 2A–C, it was found that photosynthetic rate increased firstly and then decreased during the three development stages of grapevine, and RR treatment could significantly reduce photosynthetic rate in most of the day in both veraison and maturity stages. As for photosynthetically active radiation (PAR), the results showed in the Fig. 2D–F, it was also revealed that its variation trend was roughly consistent with the photosynthetic rate, and RR treatment group was higher than the control group in most time during three development stages. As for intercellular CO_2_ concentration, we discovered that the change trends of the two treatment groups were the same, showing a decrease at first and then an increase, and there was no significant difference between RR treatment and control group in most stages and times (Fig. 2G–I).

**Figure 3:**
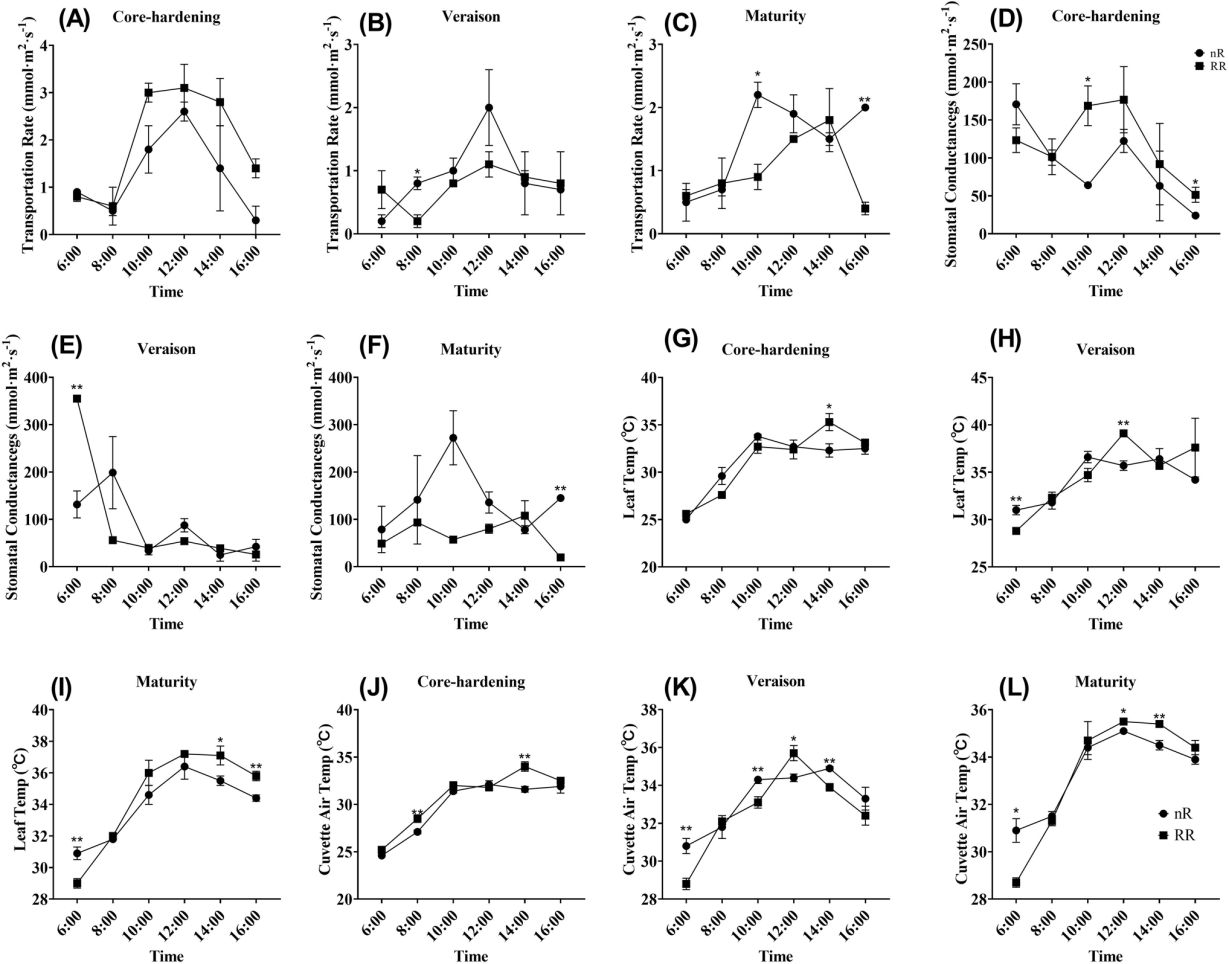
The variation tendencies of transpiration indicators with RR treatment and control in core-hardening, veraison and maturity stages. **(A–C)** Transpiration rate in three developmental stages; **(D–F)** stomatal conductance in three developmental stages; **(G–I)** leaf temp in three developmental stages; **(J–L)** cuvette air temp in three developmental stages. Data from the control group were presented as circles and data from the RR treatment were presented as square. nR indicated the control group, while RR indicated the root restriction group, * indicated the significant differences between treatments, while ** indicated extremely significant differences between treatments.

In order to further study transpiration, which is closely related to photosynthesis, we also measured four related parameters of transpiration, and the results showed that the transpiration rate of the two treatment groups showed consistent changes in the three development stages, which was increased first and then decreased (consistent with the change trends of photosynthetic rate). Meanwhile, we found that RR treatment was lower than nR treatment in the other two development stages except for that nR treatment was lower than RR treatment in the core-hardening stage (Fig. 3A–C). As for stomatal conductance, we found that it also showed a trend of first increasing and then decreasing, while only difference with the photosynthetic rate was that RR treatment was higher than the control group during core-hardening periods**,** and was lower during other two stages (Fig. 3D–F). Leaf temperature and cuvette air temperature are key indicators reflecting the strength of leaf transpiration and leaf state. As can be seen from the Fig. 3G–L, the two temperatures showed a relatively consistent trend of change, rising first and then falling, and basically reached the peak at 12:00, and RR treatment was higher in most of the time at three developmental stages.

### Analysis of different phytohormones contents

**Figure 4:**
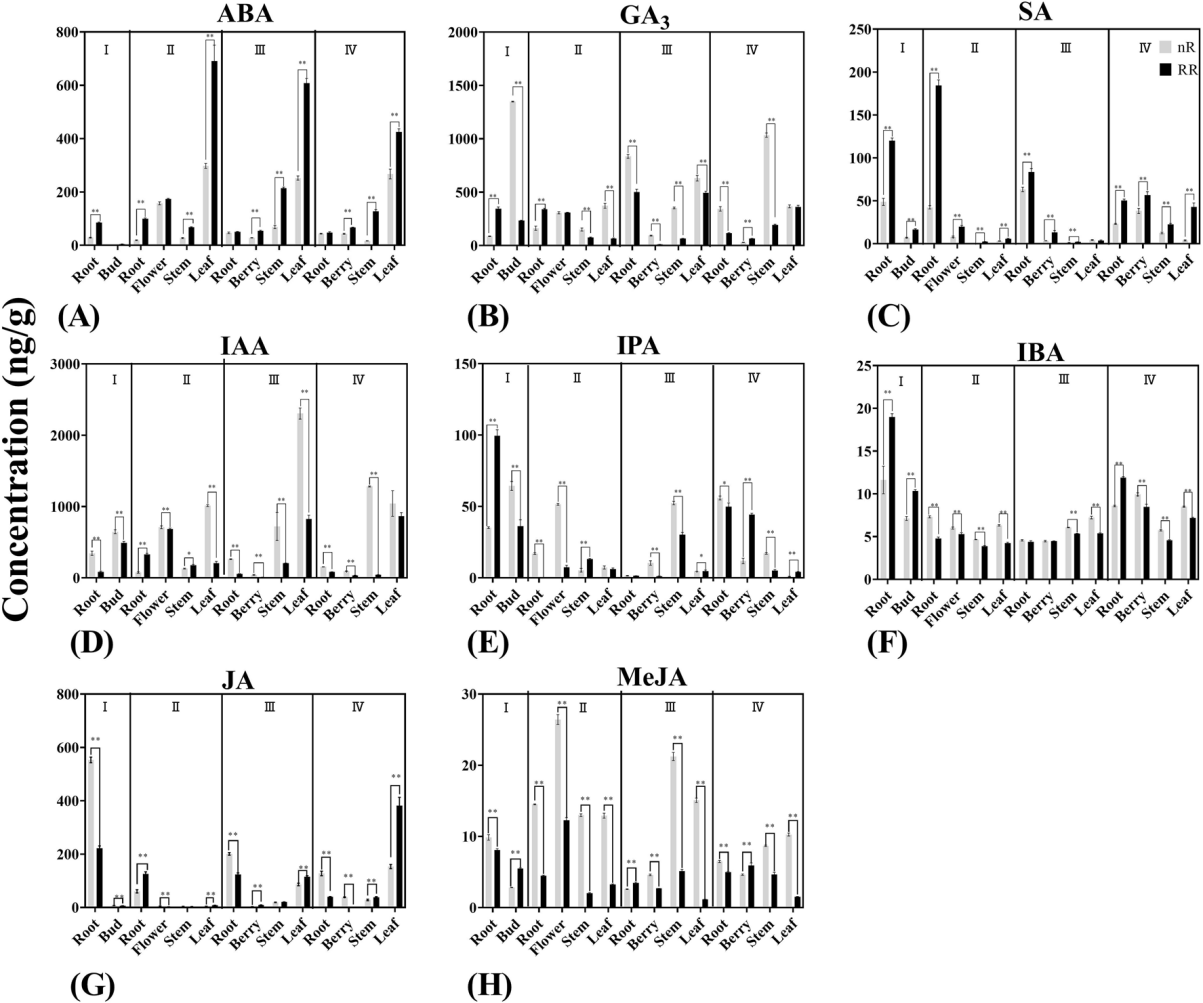
The variation tendencies of plant endogenous hormone contents (ABA, GA_3_, SA, IAA, IPA, IBA, JA, MeJA) during four developmental stages (I: Budbreak; II: Blossom; III: Veraison; IV: Maturity) in six different organs (bud, root, flower, berry, stem and leaf) with RR treatment (black) and the control (grey). * shows the significant differences with Ducan-test (p < 0.05, n = 3).

Throughout the growth and developmental period of grapevine, different types of phytohormones will undergo a series of changes, leading to the huge differences in variation tendencies of their contents. According to the previous study, the concentrations of phytohormones such as ABA, SA and JA could be significantly increased under RR treatment^41^. To gained more insight into the variation tendencies of different types of hormones in growth and development of grapevine under RR treatment, 13 different phytohormones were quantified in different grapevine organs and tissues during different developmental stages. As the results shown in Fig. 4A, the concentrations of ABA with RR treatment were significantly higher than with the control group during the stages of budbreak in root, of blossom in root, stem, and leaf, of veraison in berry, stem, and leaf, of maturity in berry, stem, and leaf. Adversely, the concentrations of IAA were significantly lower under RR treatment than the control group during the stages of budbreak in root and bud, of blossom in flower, leaf, of veraison in root, berry, stem and leaf, of maturity in root, berry and stem. Interestingly, IAA concentrations during the stages of blossom in root were significantly higher with RR treatment compared with in control group as Fig. 4D demonstrated, indicating that there were more active small cells and denser cytoplasm in the roots at blossom stage, which facilitated their division and differentiation. IBA and IPA are both important precursors in IAA biosynthetic pathways which means their levels could have direct impact on IAA concentration. As shown in the Fig. 4F, IBA concentrations roughly had the same variation tendency with IAA levels, its content was significantly lower with RR treatment than in control group in most organs and tissues during the whole developmental period. In addition, IPA concentrations shown in the Fig. 4E had revealed that its concentrations under the RR treatment during the stages of budbreak inroot, of blossom in stem, of maturity in berry and leaf were significantly higher than with the control group, while in the control group during the stages of budbreak in bud, of blossom in root and flower, of veraison in berry and stem, of maturity in stem were significantly higher compared with the RR treatment group. As shown in the Fig. 4B, the concentrations of GA_3_ had the same variation tendency with IAA either. During stages of budbreak in bud, of blossom in stem and leaf, of veraison in root, berry, stem and leaf, of maturity in root, stem, GA_3_ concentrations were significantly higher in control group than with RR treatment, while during budbreak in root and maturity in berry, GA_3_ concentrations were significantly higher with RR treatment than in control group. SA has the same function in increasing grapevine stress resistance similarly with ABA. As shown in the Fig. 4C, SA concentrations were significantly higher with RR treatment during all growth stages in all organs compared with the control group. Interestingly, the variation tendency of SA concentrations was opposite to the variation tendency of ABA concentrations, indicating these two phytohormones might play a role at different stages of grapevine development. JA also has impact on increasing grapevine resistance to diseases, RR treatment could significantly increase JA concentration compared with control treatment^43^. As shown in the Fig. 4G, JA concentrations in the RR treatment group and the control group showed opposite results. During the stages of budbreak in root, of blossom in flower, of veraison in root and of maturity in root and berry, its concentrations with RR treatment were significantly lower than with control treatment, while other developmental periods in different organs showed the inverse results, as the previous studies indicated RR treatment could significantly develop the JA concentrations along with the increasing ability of disease resistance^43^. MeJA as an organic compound, is widely generated in plants and plays important role in the induction of chemical defenses and improvement of stress resistance. As shown in the Fig. 4H, the change trend of MeJA concentrations showed the partial same variation tendency with JA. MeJA concentrations with the treatment of RR were significantly lower than with the control group in different organs during different developmental stages, except in the stages of budbreak in bud, of veraison in root and of maturity in berry.

**Figure 5:**
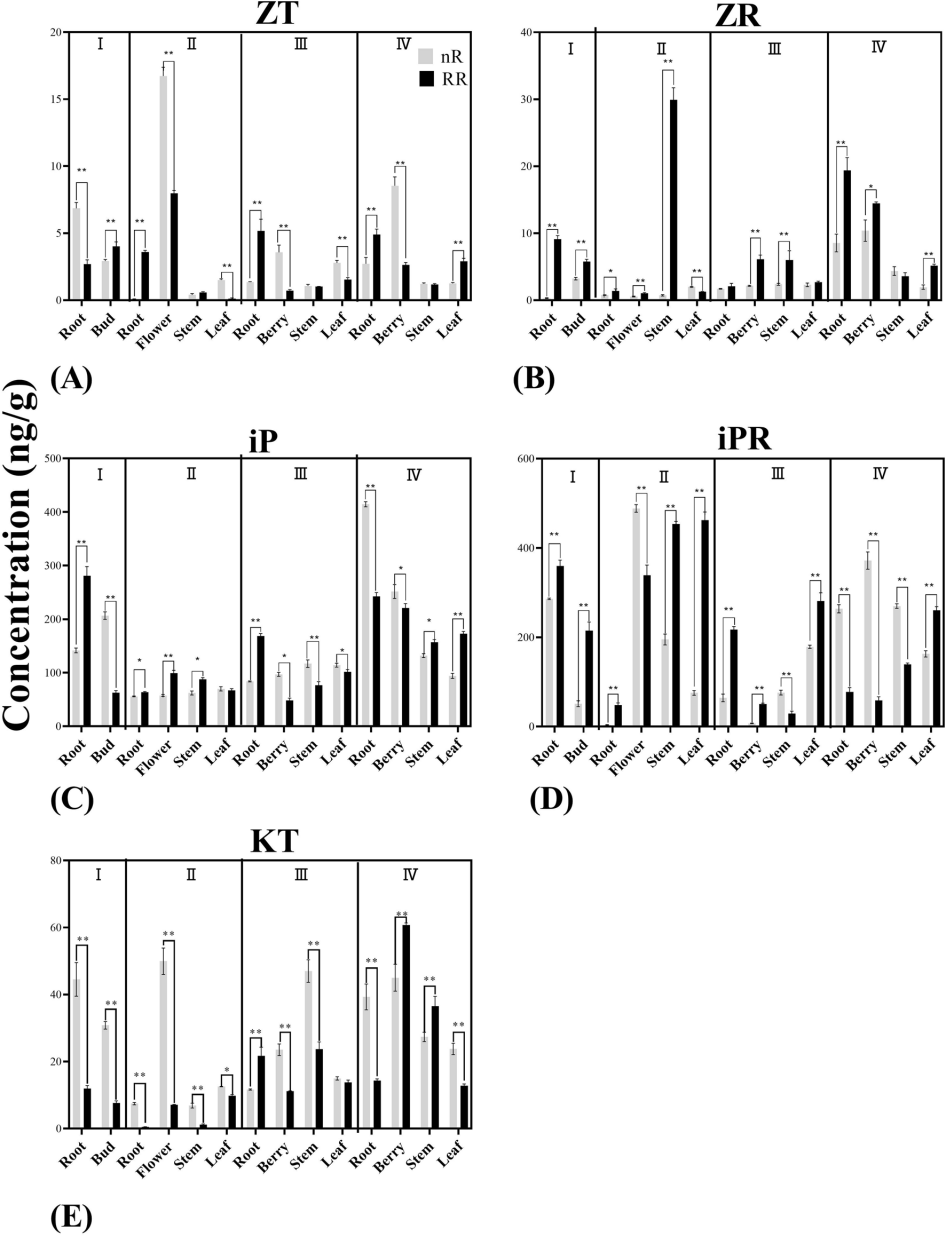
The variation tendencies of different plant endogenous hormone contents (ZT, ZR, iP, iPR, KT) during four developmental stages (I: Budbreak; II: Blossom; III: Veraison; IV: Maturity) in six different organs (bud, root, flower, berry, stem and leaf) with RR treatment (black) and control (grey). * shows the significant differences with Ducan-test (p < 0.05, n = 3).

Cytokinins (CTKs) play an important role in the growth and development of plants whole lives. In order to further explore the effect of RR treatment on the variation tendencies of this types of phytohormones in grapevine, five types of CTKs including zeatin (ZT), zeatin riboside (ZR), N-6 isopentenyl adenine (iP), N-6 isopentenyl adenine nucleoside (iPR) and kinetin (KT) were analyzed by HPLC. As shown in the Fig. 5A, ZT concentrations with RR treatment were significantly lower than in the control treatment during the stages of budbreak in root, of blossom in flower and leaf, of veraison in berry and leaf, of maturity in berry. Adversely, different results were shown during four developmental stages in root as well as during the maturity in leaf: ZT concentrations with RR treatment were higher than with the control group. ZR is an important precursor in ZT biosynthesis pathway, as shown in the Fig. 5B, its concentrations under RR treatment were significantly higher than in the control group during the stage of budbreak in root and bud, the stage of blossom in root, flower and stem, the stage of veraison in berry and stem, and the stage of maturity in root, berry and leaf. IP and iPR are also important CTKs playing a vital role in developing plant growing and cells division, as shown in the Fig. 5C,D, the concentrations of iP with RR treatment were significantly lower than in the control group during the stages of budbreak in bud, of veraison in berry, stem and leaf, of maturity in root and berry. In different organs at other stages showed different results, displaying its concentrations with RR treatment were significantly higher than in the control group. The concentrations of iPR had similar variation tendency with iP, indicating that iPR would have synergistic effect with iP. During the stages of budbreak in root and bud, of blossom in root, flower, stem and leaf, of veraison in root, berry and leaf, of maturity in leaf, its concentrations were significantly higher with RR treatment than with control treatment. Interestingly, during other stages in different organs the concentrations were significantly decreased under RR treatment. KT, as the first types of cytokinin isolated from maize plan, could significantly improve the growth of cells. Figure 5E revealed that KT concentrations were significantly lower with the RR treatment than the control group during the stages at budbreak in root and bud, at blossom in root, flower, stem and leaf, at veraison in berry and stem, at maturity in root and leaf, while at other stages in different organs showed inverse results.

### Quantification of genes in ABA, IAA, SA biosynthesis and metabolic pathway

**Figure 6:**
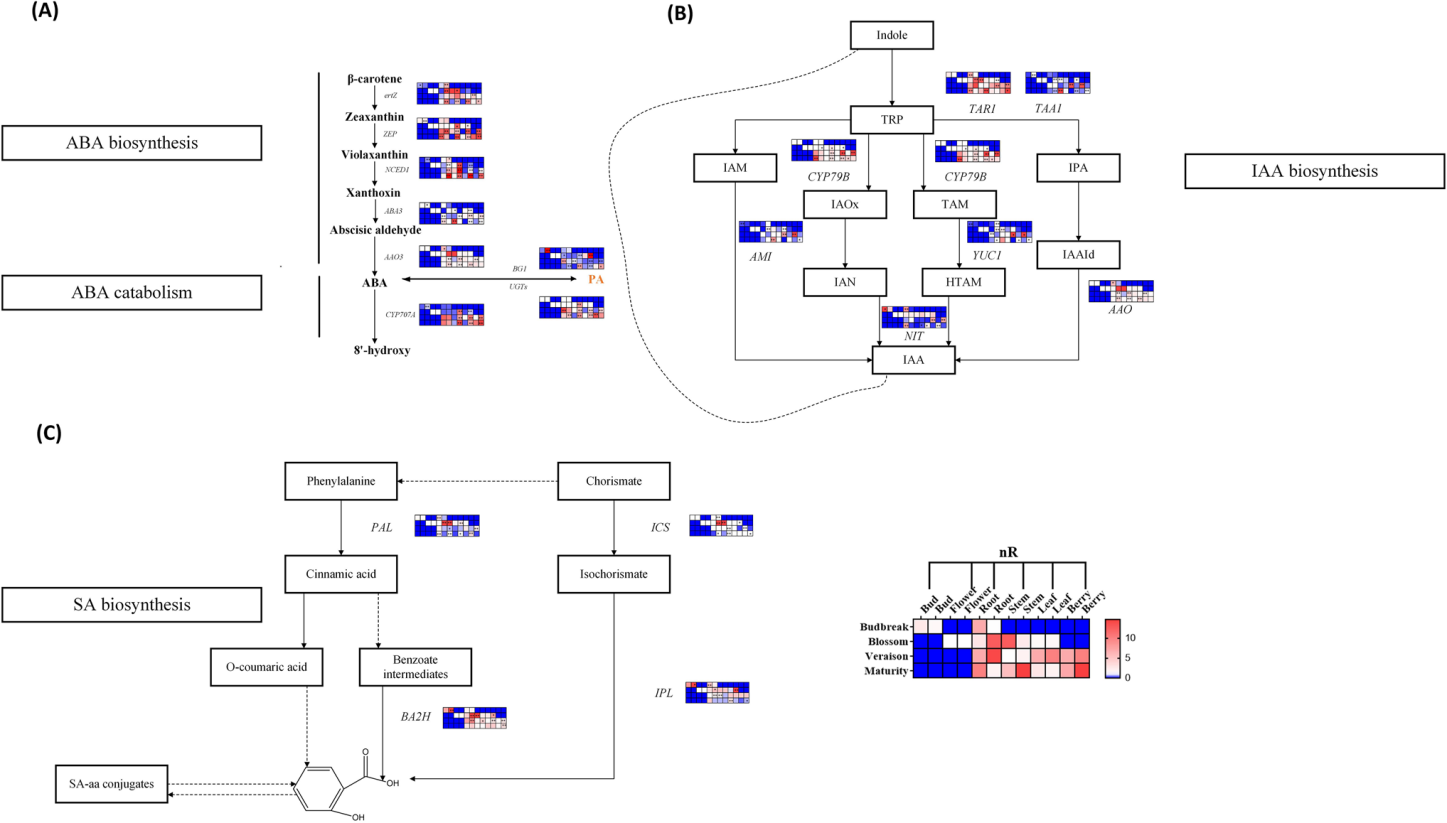
Expression levels of genes in ABA biosynthetic and metabolic pathways, IAA biosynthetic pathway and SA biosynthetic pathway. In both treatment groups, the relative expression changes of all organs in all sampling stages were log2 times of that in flower at veraison stage. Key genes (*ertZ*, *ZEP*, *NCED1*, *ABA3*, *AAO3*, *CYP707A*, *BG1*, *UGTs*, *TAR1*, *TAA1*, *CYP79B*, *AMI*, *YUC1*, *AAO*, *NIT*, *PAL*, *ICS*, *BA2H* and *IPL*) expression levels were reflected as heat map. * Indicates a significant difference between the two treatments, while ** indicates an extremely significant difference between the two treatments. **(A)** ABA biosynthesis and metabolic pathway. **(B)** IAA biosynthesis pathway. **(C)** SA biosynthesis pathway.

In order to further explore the expression of key genes in plant endogenous hormone biosynthesis pathway under root restriction, we measured the expression levels of 19 genes in ABA, IAA, SA biosynthesis and metabolic pathways, results were showed in the Fig. 6. As Fig. 6A showed, we found in the early stage of budbreak, the expression of genes related to the ABA biosynthesis (*ertZ*, *NCED1*, *ABA3*, *AAO* and *UGTs*) in bud under both root restriction and control group were low, while in the stages of blossom, veraison and maturity, in all organs most of these genes were expressed at a high level, we also found that most of the genes related to ABA biosynthesis at veraison and maturity stages were significantly increased under root restriction restriction compared with the control group. And especially in maturity stage of stem, these genes related to ABA biosynthesis were greatly increased under root restriction. Meanwhile, we found genes related to ABA metabolism (*CYP707A*) were significantly up-regulated at blossom stage, veraison stage and maturity stage in root, leaf and berry, while *BG1* confirmed to regulate ABA metabolism was down-regulated in root at blossom, in root and stem at veraison stage, and in all organs at maturity compared with the control group the expressions were up-regulated. Above findings strongly argued that root restriction could up-regulate the expression of most genes related to ABA biosynthesis and down-regulate the expression of genes related to ABA metabolism, ultimately leading to a significant increase in ABA content in all organs compared with the control group. As for the expression of genes in IAA biosynthesis pathway, the results shown in the Fig. 6B indicated that upstream genes in IAA biosynthesis pathway (*TAR1*, *CYP79B*) at veraison and mature stages in most organs were characterized by the trend of increase, and root restriction treatment could promote the improvement of these genes expression, and downstream genes in IAA biosynthesis pathway (*AMI*, *YUC1*, and *NIT*) at veraison stage and maturity stage under root restriction expression than the control group significantly reduced, while *AAO* gene expressed at a high level in RR treatment group than the control group at veraison and maturity stages, and this phenomenon could be reflected in most organs. The above results successfully indicated that although the expression of upstream genes of IAA biosynthesis was up-regulated under root restriction, downstream genes regulating IAA biosynthesis were down-regulated in most organs under root restriction, ultimately leading to the decrease of overall IAA content. Further studies on the gene expression of SA biosynthesis pathway revealed that the genes related to SA biosynthesis showed a relatively consistent change trend in all organs at all sampling stages, that was, root restriction could significantly promote the expression of genes (*PAL*, *ICS*, *IPL* and *BA2H*) in most organs at most four sampling periods, especially at veraison and maturity stages (Fig. 6C), and finally led to the increase of SA content in all organs.

### Correlation analysis between phytohormones and physical parameters

**Figure 7:**
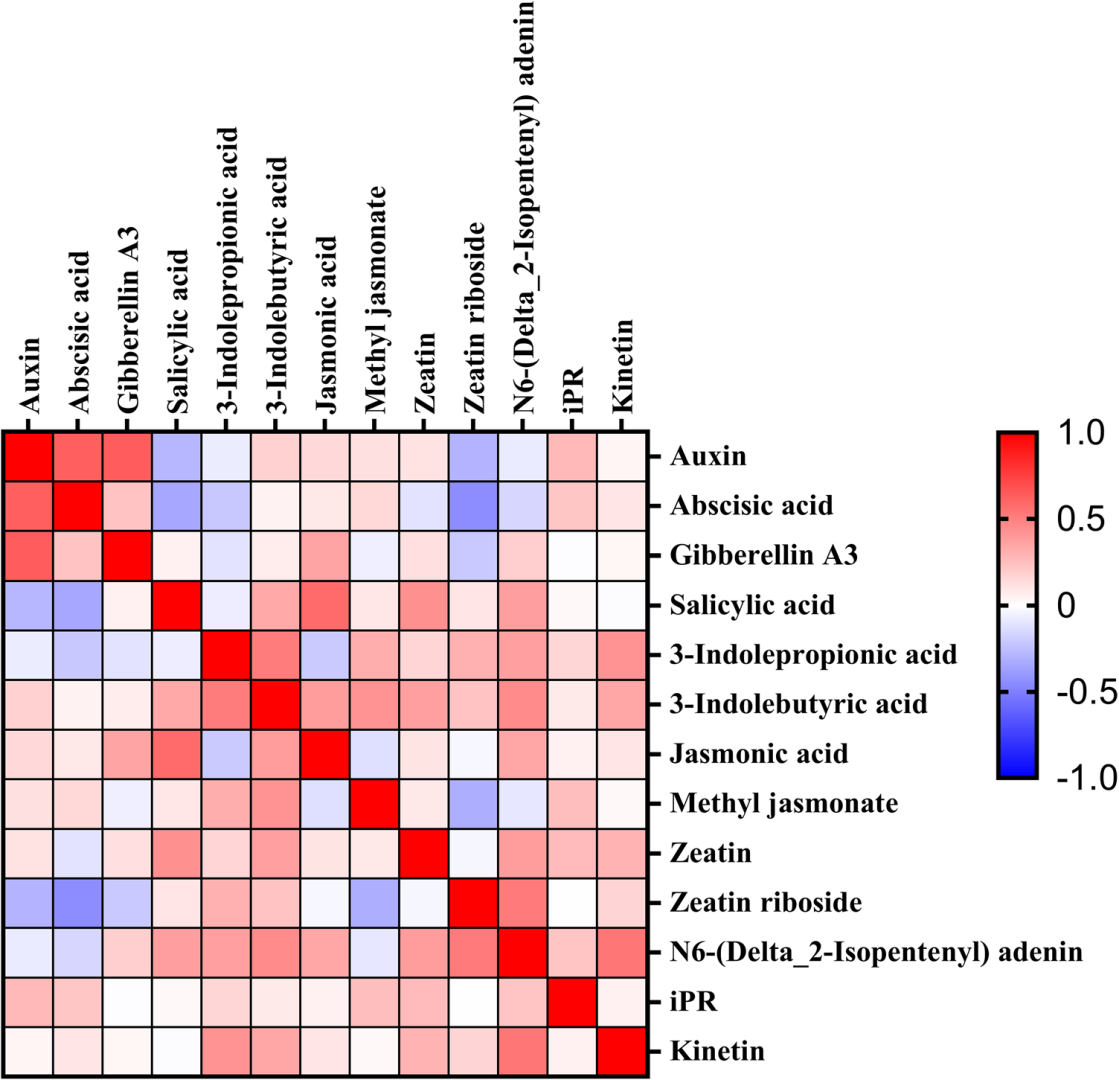
Heatmap of phytohormones concentrations in *‘Muscat Hamburg’* grape during developmental stages. A good correlation between hormones is represented by red while a poor correlation between hormones is represented by blue.

Figure 7 displayed the correlation of different phytohormones contents during different developmental stages in different organs, we discovered that KT, IBA and iPR were positively correlated with other phytohormones, and IAA and ABA showed a strong negative correlation with SA. Above results suggested that there existed synergistic and antagonistic effects between phytohormones, which jointly regulated plant growth and development, maturation and senescence. In conclusion, at different stages of grape development and ripening, the contents of various phytohormones were constantly changing in different organs due to different physiological effect. We inferred that the interaction of various phytohormones mediated the generation of other metabolites (sugars, organic acids, amino acids, fatty acids, flavonoids, phenolic substances, etc.), and eventually led to the improvement of grape quality.

**Figure 8:**
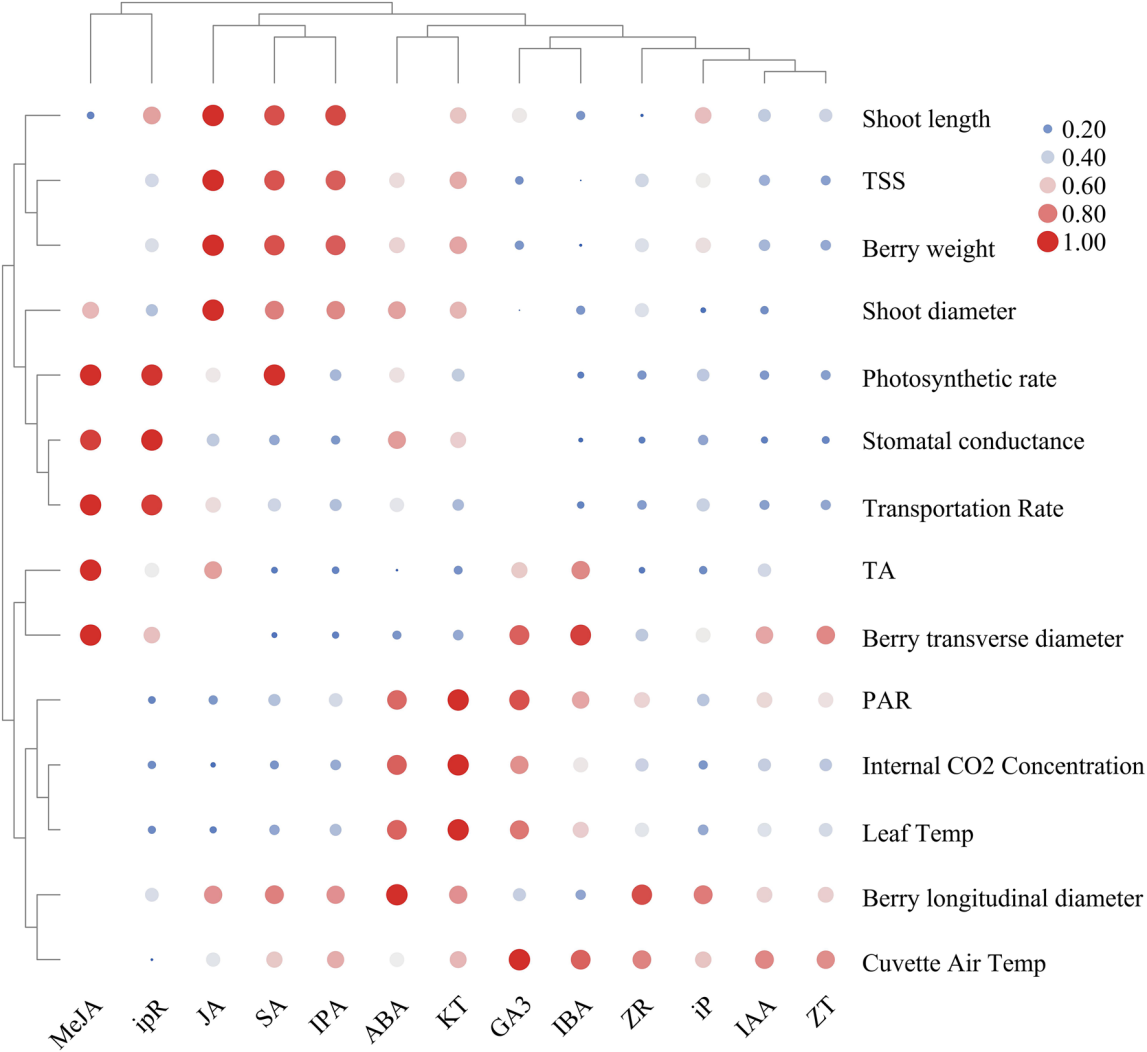
Bubble diagram of phytohormones and physical parameters in *‘Muscat Hamburg’* grape. Red indicated that phytohormones were positively correlated with physiological parameters, and blue indicated that phytohormones were negatively correlated with physiological parameters. The size of the bubble indicated the strength of the correlation, and the larger the bubble, the stronger the correlation was.

Figure 8 displayed the correlation of different phytohormones and diverse physical parameters, we found that different phytohormones had different contributions to different physiological parameters. By cluster analysis, we divided MeJA and iPR into a class, divided JA, SA and IPA into a class, divided ABA, KT and GA_3_ into a class, divided IBA, ZR, iP, IAA and ZT into a class. In grapevine, there existed strong correlation between MeJA, iPR and transpiration, photosynthesis; strong correlation between JA, SA, IPA and shoot length, TSS, berry weight, shoot diameter; strong correlation between ABA, KT, GA_3_ and PAR, internal CO_2_ concentration, leaf temp; While strong correlation between IBA, ZR, iP, IAA, ZT and cuvette air temp. Although our results demonstrated that different phytohormones have different contributions to grape plant growth and development, photosynthesis and fruit quality related parameters, they ultimately promoted the overall improvement of grape growth, physiological parameters and quality parameters.

## Discussion

As is widely acknowledged that phytohormones play important role in the whole growth and development of different plants. Through many years of exploration, it has been identified five main kinds of plant endogenous hormone including IAA, GA, ETH, CTK, and ABA^1^. Nowadays, more than 20 types of phytohormones had been extracted from different organs and tissues in plants along with the revelation of their physiological functions and actional mechanism. With the development and progress of scientific technology, hormonal biosynthesis and hormonal transduction pathways have been clarified.

IAA is one of the first phytohormones isolated from living plant tissues, it has been reported that the polar transport of IAA is closely related to plant growth and development, it could stimulate cell elongation, promote vascular bundle differentiation, control apical dominance and regulate flowering and fruit-setting of plants^6^. Due to the transport of IAA, the content of IAA varies in different development stages and organs of grapevine. In terms of IAA concentration, our results revealed that RR treatment could significantly decreased its concentrations in most stages and organs, indicating that RR treatment could inhibit the growth of grapevine, which led to grapevine phenotypes changed including shorter stem length or smaller berry vertical and horizontal diameter, these finding was consistent with previous studies implying that RR treatment could reduce IAA concentration in grapevine^43^. Thus, we hypothesized that RR treatment affected the biosynthesis and distribution of IAA and inhibited grape growth by slowing down the transport of IAA, resulting in the dwarf of grapevine and the shorten of new tips. ABA is another widely studied plant endogenous hormone which has been demonstrated to play important role in accelerating abscission of leaves and berries^8^, dormancy of buds^9^, formation of stress resistance^9^, regulation of sucrose transporter expression^44^, formation of anthocyanin^45^, regulation of stomatal opening^10^. Our results implied that RR treatment could significantly develop its concentrations during most growth stages especially in berries and leaves consistently with the previous studies^43^. We deduced that RR treatment could develop ABA content and correspondingly develop grapevine stress resistance. Earlier studies focused on physiological responses implied GAs could significantly promote elongation of stems and germination of seeds as well as acceleration of blossom^13,46^. It could be implied in our results that RR treatment could significantly reduce GA_3_ concentrations during most growth stages in different organs accordantly with previous studies^43^. The results also confirmed that GA_3_ might generate during the veraison stage and indirectly promoted grapevine growth and berry enlargement. We also found GA_3_ concentrations were higher in root and leaf than other organs, directly confirming the biosynthesis sites of GA might mainly concentrated in root or leaf. SA, a phenolic derivative, have been confirmed to play important role in plant growth, thermogenesis, flower induction as well as stress and disease resistance^27^. Our results implied that RR treatment could significantly increase SA contents during all stages especially in root and berry, which were consistent with previous studies^43^. We deduced that RR treatment could increase SA concentration to improve grapevine’s ability of resisting stresses and diseases. JA, a plant endogenous hormone found in higher plants, could regulate stomatal opening, rubisco biosynthesis, glucose transportation and disease resistance formation^17^. Our results showed that the variation tendency of MeJA contents in different organs during different developmental stages were partial consistent with JA contents, uncovering that MeJA might have a synergistic effect with JA in growth regulation as well as stress resistance improvement of plants. It had been sufficiently revealed that CTKs could promotes cell division, control cell differentiation and regulated vascular cambium development^47,48^. Through measuring 5 types of CTKs, our results revealed that during different stages CTKs concentration showed significantly differences. ZT concentrations with RR treatment were significantly lower than the control group in berry and flower while higher in root and leaf. Then we deduced that ZT would be generated or be transported into root and leaf more. ZR concentrations showed completely different results, indicating RR treatment could significantly increase its contents. Based on the results, we deduced that ZR and ZT had antagonistic effects and played different roles in regulating grape growth and development. Meanwhile, iP concentrations with RR treatment were significantly higher than with the control treatment during the stages of blossom in all organs and of maturity in stem and leaf. We deduced that RR treatment could regulate iP transporting direction and lead to variation of distribution location as well as different content. Interestingly, iPR concentrations showed the same variation tendency as iP concentrations, it could be inferred that iP played synergistic role in regulating grapevine growth with iPR. Our results also revealed that KT concentrations under RR treatment were significantly decreased especially during blossom and maturity in leaf, stem and root. Therefore, we deduced that RR treatment could shorten new-tip length and slow grapevine growth by reducing KT concentration.

Root restriction, as a used cultivation technique in agriculture, by confining roots to a fixed space and limiting the plant's growth potential, fruit quality is improved (sugar accumulation, anthocyanin enrichment) despite reduced plants photosynthesis^43,49^. In addition, this cultivation method could also significantly influence phytohormones levels during the whole cultivation period. In our study, we found that under RR treatment, the growth of grapevine was inhibited and photosynthesis decreased, while the sugar content significantly increased and titratable acid content decreased, accompanied by the differences of endogenous hormones in various organs. The present results also showed that a closely relationship between the hormones was existed, we deduced that under the root restriction treatment, the contents of hormones that promoted plant growth including IAA, GA_3_, CTKs, JA as well as MeJA showed the mostly consistent trends in the whole developmental stages in different organs revealing that they might have synergistic effect in promoting plant elongation and berry ripening. On the contrary, the concentrations of SA and ABA were significantly enhanced with the RR treatment along with the improvement of stress resistance. Therefore, we deduced that phytohormones such as SA and ABA that enhanced plant stress resistance would respond to root restriction, resulting in the increase of their contents. Each hormone was bound to pass through a series of signal transduction and crosstalk affecting the expression of genes on their synthetic pathway, finally resulting in differences in hormone contents. Our studies aimed to investigate the variation tendency of phytohormones during four stages (budbreak, blossom, veraison, maturity) in different organs (bud, root, berry, flower, stem, leaf) between RR treatment and the control treatment. Finally, based on the obtained results, we concluded that RR treatment could indeed extremely influence the concentration of several phytohormones. To further support the conclusion concluded by us about the change trend of ABA, IAA and SA content under root restriction, key genes expression in ABA, IAA and SA biosynthesis pathway were determined, the experimental results showed that most of the genes involved in ABA biosynthesis under root restriction at veraison period and maturity period of most organs were significantly up-regulated, while genes related to its metabolism were also slightly upregulated, downstream genes related to IAA biosynthesis were down-regulated at most stages and in most organs under root restriction treatment. In addition, downstream genes (*BA2H*) in SA biosynthesis pathway were significantly up-regulated in the root restriction group at both veraison stage and maturity stage. Above results were consistent with previous study^43^, further confirmed that root restriction had a great impact on ABA, IAA and SA biosynthesis, which was not only reflected in the changes of ABA, IAA and SA contents, but also in the differences of gene expression in their biosynthesis and metabolic pathway. The correlation between phytohormones and grape physiological parameters has not been systematically studied, to further study the correlation between phytohormones and physiological parameters under root domain restriction, we also carried out correlation analysis. The results demonstrated that different phytohormones contributed different on the change of physiological parameters in the process of grapevine growing, there exist correlation between MeJA, iPR-photosynthesis and transpiration; JA, SA, IPA-berry weight, TSS; ABA, KT, GA3-PAR and leaf temperature; IBA, ZR, iP, IAA, ZT-Leaf cuvette air temperature and berry size.

In conclusion, root restriction could significantly influence the contents of phytohormones. In particular, the contents of ABA and SA were increased and the contents of IAA were decreased, and most genes of ABA and SA biosynthesis pathway were up-regulated in most organs at various sampling stages, while the downstream genes of IAA biosynthesis pathway were down-regulated in most organs. Through further correlation analysis, it was concluded that different phytohormones contributed differently to the change trend of physiological parameters (photosynthesis, transpiration, growth, fruit quality) of grapevine. The results provided data support for rapid detection and quantitative analysis of phytohormones in grape under root restriction cultivation. However, further researches on mechanisms of phytohormones interaction and regulation of key genes and transcription factors in phytohormones biosynthesis pathway under root restriction treatment need to be implemented.

## Materials and methods

### Materials collected

**Figure 9:**
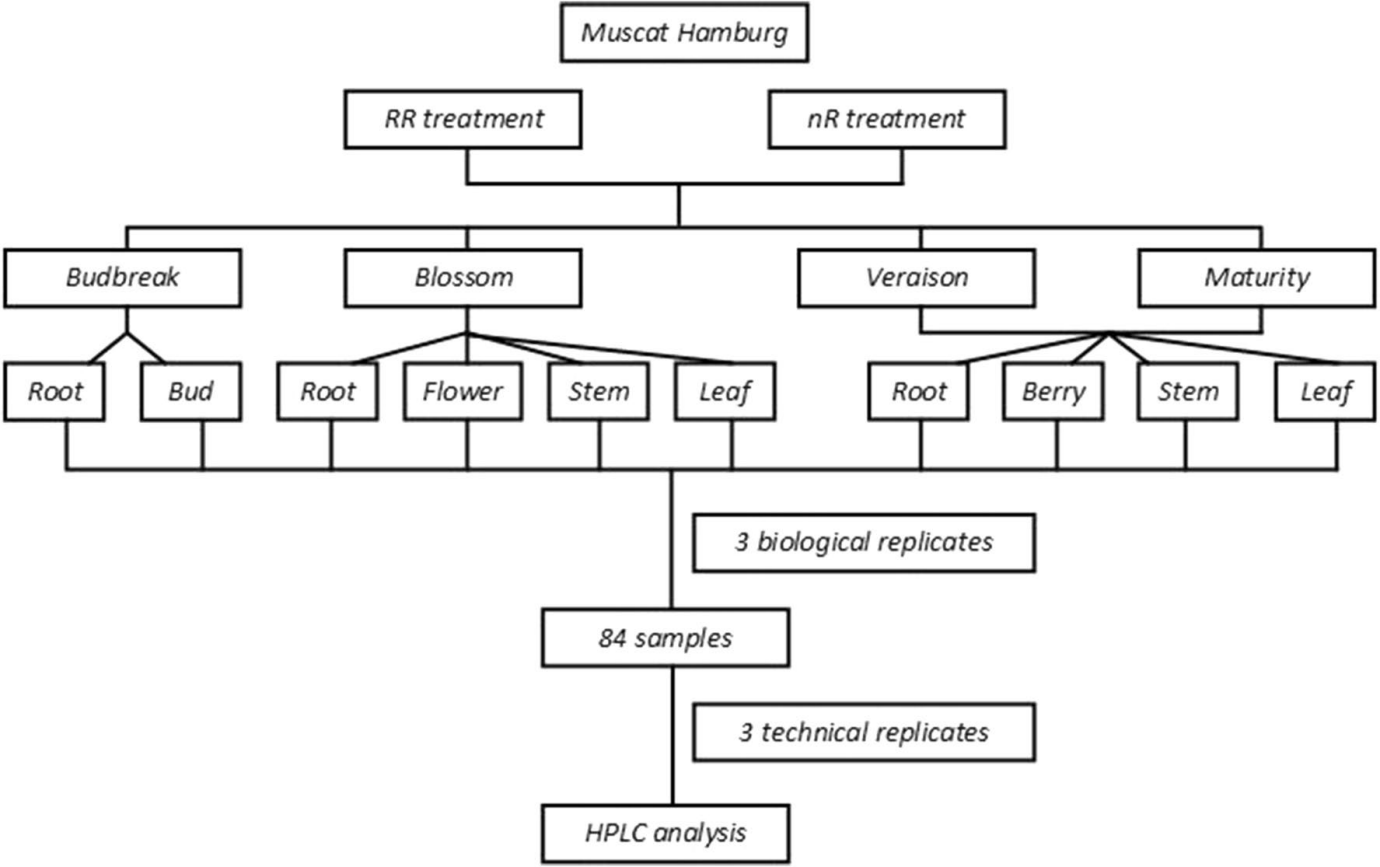
Technical route of this experiment.

This experiment was established during the year of 2019 to 2020 in the experimental greenhouse located in Shanghai Jiao Tong University, Shanghai, China (31°11′N, 121°29′W), using five years old ‘*Muscat Hamburg’ (Vitis ‘Muscat of Alexandria’* × *Vitis ‘Trollinger’)*. The grapevines applying with RR treatment were planted in a spacing of 1.5 m × 2.0 m in north–south oriented rows in a 40 cm deep cultivation soil, while the grapevines cultivated in the control group were only placed in cultivation soil and not limited root growth. Both two groups were applied to the same water and fertilizer condition. This experiment respectively collected buds, roots, flowers, berries, stems and leaves from seven grapevines in four different developmental stages including the budbreak stage (I), the blossom stage (II), the veraison stage (III) and the maturity stage (IV). According to the collecting stages, twenty-eight grape clusters without visible pests and diseases were randomly picked from seven ‘*Muscat Hamburg’* grapevines and immediately transported back to laboratory within 2 h. Flowers of blossom stage (II), fruits of veraison (III) and maturity stages (IV), buds of budbreak stage (I) as well as stems and leaves of blossom (II), veraison (III) and maturity (IV) stages without damage were selected to be prepared for physiological parameters measurements, and then immediately grinded by nitrogen and frozen in −80 °C refrigerator for further studies. The specific technical route was shown in Fig. 9.

### Physiological parameters measurement

Different physiological parameters, such as berry weight, longitudinal and transverse diameter, shoot length and diameter, total soluble solid (TSS), photosynthetic rate and stomatal conductance were measured during the veraison of ‘*Muscat Hamburg*’ *(Vitis ‘Muscat of Alexandria’* × *Vitis ‘Trollinger’)*. Twenty shoots were randomly picked from each RR treatment group and the control group on fourteen grapevines with the measurement of shoot lengths and diameters using tapeline in the specific sampling times (shown as DAF). In the same time, twenty berries without damage from each group on fourteen grapevines also were selected to prepare for the next experiments. Approximately 50 grape berries without pedicels and seeds were squeezed into grape juice for further researches. Berry weights were measured by analytical balance (Sartorius, Germany), longitudinal and transverse diameters were measured by vernier caliper (Mitutoyo, TKY, Japan), total soluble solid (TSS) was measured by refractometer (OWELL, Hangchow, CHN), titrating acid (TA) was measured by potentiometric titrator (HOGON, CHN), and photosynthetic rate, stomatal conductance, photosynthetically active radiation (PAR), internal CO_2_ concentration, transpiration rate, leaf temperature, cuvette temperature were quantified by CIRAS-3 portable photosynthetic fluorescence measurement system (Hanshatech, CA, USA) at core-hardening, veraison and maturity stages. All tests were implemented for more than three technical duplicates or three biological replicates.

### IAA, ABA, SA, IPA, IBA, GA_3_, JA and MeJA quantification

For the quantification of IAA, ABA, SA, IPA, IBA, GA_3_, JA and MeJA, the experiment was performed according to the previous methods with a little adjustment^42^: Firstly, 0.1 g of sample was dissolved in the 1 ml Mod-Bielesk solution (Methanol: Formic acid: ddH_2_O = 15:1:4), then placed in −20 °C for a night, next mixed liquor was centrifuged using high speed centrifuge (CENCE, CHN) under the condition (4 °C, 13,000 r/min) for 20 min, and then supernatant was collected and extracted by 0.5 ml Mod-Bielesk, next shocked about 5 min for 2 times and centrifuged again (4 °C, 13,000 r/min) to collect liquid supernatant. Then solution should be extracted by CNWBOND HC-C18 SPE Cartridge (CNW, German) and filter liquor was evaporated by rotary stem evaporator (RE, SHH, CHN) at 40 °C, then promptly redissolved by 5 ml of 1 M formic acid. Next, solution should be extracted by Poly-Sery MCX SPE Cartridge (CNW, German) and then 5 ml of 1 M formic acid was added to wash the column (W1), finally eluted by 5 ml methanol (W2) and redissolved in 0.5 ml extraction solution (Methanol: Isopropanol: Acetic Acid = 20:79:1) after eluant was condensed by rotary stem evaporator at 40 °C (RE, SHH, CHN). Etraction solutions containing IAA, ABA, SA, IPA, IBA, GA_3_, JA and MeJA were prepared for analyzation by HPLC. Standard substances of IAA, ABA, SA, IPA, IBA, GA_3_, JA and MeJA (Anpel, SHH, CHN) dissolved in the same extraction solution (Methanol: Isopropanol: Acetic Acid = 20:79:1) with different concentrations were also prepared for analyzation by HPLC.

Chromatographic separation was performed in the LC3000 Semi-preparation Isocratic HPLC System (CXTH, BJ, CHN) by a Capecell PAK C18 column (4.6 mm × 50 mm, 1.8 µm) with mobile phase A (0.01% formic acid–methanol) and mobile phase B (0.01% formic acid–water), with an injection volume of 0.5 μ and the flow rate of 0.35 ml/min (IAA, IPA, IBA and GA_3_), 0.8 ml/min (ABA), 1.5 ml/min (SA, JA and MeJA). The gradient program was as follows: 0–4 min, 20% A; 4–8 min, 20% A -50% A; 8–20 min, 50% A-80% A; 20–22 min, 80% A; 22–22.2 min, 80% A-20% A; UV wavelength was set to 254 nm and temperature of chromatographic column was adjusted to 40 ℃. All reagents (CNW, German) used for HPLC were chromatographic grade, being filtered through 0.22 μm membrane and ultrasonic treated (200 Hz, 30 min) for more than twice.

### ZT, ZR, iP, iPR and KT quantification

After the Poly-Sery MCX SPE Cartridge (CNW, German) had been eluted by methanol, the column should be washed again by volume of 5 ml 0.35 M ammonia (E1) and then eluted by the volume of 5 ml 0.35 M ammonia in 60% methanol (E2), finally filter liquor should be condensed by rotary stem evaporator (RE, SHH, CHN) at 60 °C and then redissolved with 0.5 ml of 5% acetonitrile. Etraction solutions containing ZT, ZR, iP, iPR and KT were prepared for analyzing by HPLC. Standard substances of ZT, ZR, iP, iPR and KT (Anpel, SHH, CHN) dissolved in the 5% acetonitrile with different concentrations were also ready for quantification in HPLC.

Chromatographic separation was performed in the LC3000 Semi-preparation Isocratic HPLC System (CXTH, BJ, CHN) by a Capecell PAK C18 column (4.6 mm × 50 mm, 1.8 µm) with mobile phase A (0.06% acetic acid water) and mobile phase B (0.06% acetic acid–methanol), then an injection volume of 0.5 μ and the flow rate of 0.5 ml/min should be set in HPLC program. The gradient program was set as follows: 0–8 min, 99% A-55% A; 8–14 min, 55% A-30% A; 14–16 min, 30% A-1% A; 16–24 min, 1% A-99% A; UV wavelength was 254 nm and the temperature of chromatographic column was 40 °C. All reagents (CNW, German) used for HPLC were chromatographic grade, being filtered through 0.22 μm membrane and ultrasonic treated (200 Hz, 30 min) for more than twice. The information of each hormone eluted by HPLC was shown in the Table 1.

**Table 1:** The information of each hormone eluted by HPLC.

Compounds	Retention time (min)	Peak area	Molecular formula	Calibration curves
Abscisic acid	12.286	7,573,628	C_15_H_20_O_4_	Y = 120550X − 6162.4
Auxin	20.46	13,852,722	C_10_H_9_NO_2_	Y = 35324X + 109,746
Gibberellin A3	17.781	2,309,297	C_19_H_22_O_6_	Y = 29314X + 38,787
Salicylic acid	10.033	175,455	C_7_H_6_O_3_	Y = 3264.3X − 14219
3-Indolepropionic acid	22.059	54,120	C_11_H_10_NO_2_	Y = 36831X − 18773
3-Indolebutyric acid	23.623	88,712	C_12_H_13_NO_2_	Y = 36604X + 59,323
Jasmonic acid	19.245	1,418,457	C_12_H_18_O_3_	Y = 17424X − 265.8
Methyl jasmonate	24.089	1602	C_13_H_20_O_3_	Y = 23484X − 5404.8
Zeatin	17.248	89,642	C_10_H_13_NO_5_	Y = 113736X + 16,113
Zeatin riboside	19.688	116,127	C_15_H_21_N_5_O_5_	Y = 78039X + 35,944
N6-(Delta 2-Isopentenyl)-adenine	22.539	6,058,588	C_10_H_13_N_5_	Y = 16766X + 2689.6
N6-(Delta 2-Isopentenyl)-adenine nucleoside	14.268	89,391	C_15_H_21_N_5_O_4_	Y = 1440.7X + 10,175
Kinetin	17.824	72,057	C_10_H_9_N_5_O	Y = 22046X + 11,778

### Real time qRT-PCR analysis

RNA extraction was carried out on RNA prep Pure Plant Plus Kit (TaKaRa, Dalian, China), which used root, stem, leaf, flower, berry, bud from grapevine in all sampling stages, Purity and completeness were analyzed using the Bio-RadXR Gel Imaging analysis system (Bio-RAD, CA, USA). Following the manufacturer's instructions, the PrimeScript™RT Kit with gDNA Eraser (Perfect Real time) (TaKaRa, Dalian, China) used 1 μg of total RNA extracted from collected samples, designed to obtain the first strand of cDNA.

The final composition volume was 10 μL, consisting of 1 μL cDNA, 5 μ L TB Green®Fast qPCR Mix, 3 μL ddH_2_O and 1 μL positive and negative primer mixture. qRT-PCR was then performed using CFX coupled with Real Time PCR detection system (Bio-RAD, CA, USA). All sequences of genes and transcription factors using in this study were downloaded from EnsemblPlants (http://plants.ensembl.org/info/about/index.html). The specific primers (55 genes, 7 transcription factors and actin) were designed using qPrimerDB (https://biodb.swu.edu.cn/qprimerdb/browse_plants), and the sequences and information of genes and transcription factors were all listed in Table S1. qRT-PCR procedure :95 °C was treated for 20 s, followed by 95 °C for 15 s, 55 °C for 15 s, and 60 °C for 15 s, with 39 cycles. The final relative expression of each gene was analyzed by 2^−△△Ct^ method.

### Statistical analysis

Results were analyzed using at least three independent replicates including the mean ± standard error (SE) by data processing system SPSS 16.0 statistical software package (IBM, Armonk, NY, USA). The method of Ducan’s multiple range test (ANOVA) was used to analyze the differences of data significance at the level of P < 0.05. Correlation analysis was carried out by SPSS, by using the difference between physiological parameters of root restriction cultivation and control group, the difference of phytohormones of the two treatment groups was compared to complete correlation analysis, and the bubble diagram was drawn by TBtools (CAN, CHN). Figures were drawn by GraphPad Prism 8.0 (GraphPad Software Inc., San Diego, CA, USA) and Visio 2020 (Microsoft, SEA, USA).

### Ethics declarations

The authors declare no competing interests. Experimental and field studies of plants (cultivated or wild), including the collection of plant material, have been conducted in compliance with relevant institutional, national and international guidelines and legislation.

## Supplementary Information

Supplementary Table S1.
