# Supplementary material for: Effects of root restriction on phytohormone levels in different growth stages and grapevine organs
Source: Sci Rep. 2022 Jan 25;12:1323. doi: 10.1038/s41598-021-04617-6 (PMC8789776; doi:10.1038/s41598-021-04617-6)
Supplement: Supplementary file 1 — Supplementary Table S1. [file 41598_2021_4617_MOESM1_ESM.docx]

**Table S1.** Primers for qRT-PCR quantification, the sequences of genes in ABA, IAA and SA biosynthesis pathways were all obtained in EnsemblPlants (http://plants.ensembl.org/info/about/index.html).

| **Target genes** | **Forward sequence（5’ → 3’）** | **Reverse Sequence（5’ → 3’）** |
| --- | --- | --- |
| *Vvactin* | GACAATTTCCCGTTCAGCAGT | GATTCTGGTGATGGTGTGAGT |
| *VvNCED1 (VIT_19s0093g00550)* | CGTGGCTCTCCTGTGGTGT | GCCTTCGTCGCATTCGTTG |
| *VvPP2C1 (VIT_13s0067g01270)* | CCACGCTTCCCTCAAACCCTCCCA | CATCCACCACTCCATCATCCCCGC |
| *VvABA3 (VIT_17s0000g02290)* | CGAGATGAATGGAGCAGGGAG | TGGACTTGGAGTCGAAGCAGA |
| *VvCYP707A (VIT_02s0087g00710)* | GTCTGAACCAACTGTCAACGA | TGAAAGGAGTGTGAGAACGCA |
| *VvAAO3 (VIT_11s0016g03490)* | AAGTGAGGTGGAGGTAAAT | ACTGCTTGGGTATGGTGTC |
| *Vvertz (VIT_02s0025g00240)* | GCTATCGTTGCTGTCTACTACA | CACAGAAAGAGCAAATGTACCC |
| *VvZEP (VIT_07s0031g00620)* | GATACAGAGTAATGCATTGGCG | CGCAGGAGTGAATGTATCAAAC |
| *VvBG1 (VIT_01s0011g00760)* | GCAATCAAGGATGGAGTTGATG | GAAACGTTTGGTGTAACCTTGA |
| *VvUGTs (VIT_05s0094g01010)* | GGATTGGTATAGGAGTTGAGGG | TCTAGCAAGCTCTTTTAGGACC |
| *VvTAA1 (VIT_17s0000g08990)* | CAAGTCTGCAAGTGAATACACC | CGTTGAAGGCACTATAAATGGG |
| *VvTAR (VIT_14s0083g00460)* | ATTTTGACCTTCCAGCCTATGA | TAGTAAATGGCTTCACCTCAGG |
| *VvCYP79B1(VIT_13s0156g00590)* | CTCTCACTTAGATCCTTCGGAC | CCCTCTGAAGTTGATAATCCGA |
| *VvYUC1 (VIT_00s0233g00120)* | TACAAGCACATTTTTCCACCAG | CGACAAGTAAAAGGCTTCAACA |
| *VvNIT1 (VIT_04s0008g01800)* | ACGGATTTCACTCCTGACTATG | TGTTCTCGAGACAGTCATTTGA |
| *VvAAO (VIT_12s0121g00400)* | CACTACCATCCCCAAAATCTCT | CACAACTTCTTCTTCTTCTGGC |
| *VvPAL (VIT_11s0016g01520)* | GAGCTGATTCGGTTTCTCAAC | TACCCTTGCATCAGAGTATTCG |
| *VvICS1 (VIT_01s0010g00900)* | AGAAGATTAAGGACGCTGTTGA | CTGAAAGAAGGACACGTAAAGC |
| *VvIPL (VIT_17s0000g05750)* | GCAGTGTTCTTCCGACATATTC | CATTAGCTCTTGGATCAAACCG |
| *VvBA2H (VIT_04s0008g01800)* | ACGGATTTCACTCCTGACTATG | TGTTCTCGAGACAGTCATTTGA |
